# Application of an iterative generative AI-augmented teaching model in medical immunology education

**DOI:** 10.3389/frai.2026.1788369

**Published:** 2026-04-13

**Authors:** Yanli Niu, Jiajin Xu, Wenqi Yang, Hongmin Yuan, Zhonghua Liu, Weijuan Zhang, Lingyun Liu

**Affiliations:** 1 School of Basic Medical Sciences, Henan University, Kaifeng, China; 2 School of Life Sciences, Henan University, Kaifeng, China

**Keywords:** clinical decision-making, four-component instructional design (4C/ID), generative artificial intelligence, immunology education, iterative teaching model

## Abstract

**Introduction:**

Immunology education faces persistent challenges, including abstract concepts, theory-practice disconnect, and delayed feedback. Generative AI offers opportunities, yet its integration into pedagogical frameworks remains underexplored.

**Methods:**

We developed an Iterative AI-Augmented Immunology Education (IAIE) model integrating generative AI with the 4C/ID model and cognitive load theory. A 12-week quasi-experimental study enrolled 177 medical undergraduates (experimental: n = 88; control: n = 89), with outcomes assessed using the Host-Pathogen Interactions Concept Inventory (HPI-CI) and a modified Clinical Decision-Making in Nursing Scale (CDMNS).

**Results:**

The experimental group showed significantly greater knowledge mastery (82.7 vs. 70.1, Cohen’s d =1.12) and clinical decision-making accuracy (+31.7%). Over 84% of students recognized AI’s value in understanding complex mechanisms. Reflective practice scores indicated high levels of reflective ability, and DREEM assessments confirmed strong student acceptance of the learning environment.

**Discussion:**

The IAIE model effectively bridges theory and practice through AI-driven scaffolding that modulates cognitive load, fostering higher-order thinking and critical engagement with AI-generated content. This theory-informed framework offers a transferable approach for competency-based medical education.

## 1 Introduction

Currently, medical education is undergoing a profound transformation from traditional knowledge transmission to the cultivation of higher-order competencies. In this context, immunology, as a core component of medical education, presents significant challenges for traditional teaching methods due to its abstract content, complex mechanisms, and close ties to clinical practice. Research indicates that students often feel confused when trying to understand complex mechanisms such as antigen–antibody interactions and immune regulatory mechanisms, and they struggle to apply theoretical knowledge learned in the classroom to real clinical scenarios (Cavuoto Petrizzo et al., 2019; Haidaris and Frelinger, 2019; Porter et al., 2021; Siani et al., 2024). Furthermore, the lack of interactive teaching has further diminished students’ motivation to learn (Stranford et al., 2020; Sannathimmappa et al., 2022). These challenges underscore the urgent need to introduce innovative teaching techniques and models in modern medical education.

The rise of generative AI technology presents new opportunities for educational innovation. Its powerful content generation and scenario simulation capabilities position it as a potential bridge between theory and practice (Yu and Guo, 2023; Lakshan et al., 2024; Wang et al., 2025). However, existing AI educational applications exhibit significant limitations. Firstly, most AI systems focus on optimizing the efficiency of knowledge transfer while neglecting the gradual development of clinical reasoning skills (Lakshan et al., 2024). Secondly, the iterative teaching process often relies on the experience of educators, lacking a data-driven dynamic optimization feedback loop mechanism (Ninaus and Sailer, 2022). Particularly in the context of immunology education, effectively integrating a deep understanding of micro-level mechanisms with the cultivation of macro-level clinical decision-making abilities remains a weak point in current technological solutions.

To address these gaps, this study proposes the Iterative AI-Augmented Immunology Education (IAIE) model. This study aims to extend the current research landscape of AI in medical education for several reasons:

(1) Different educational contexts: Bridging the micro–macro gap in immunology education. While existing technological solutions have primarily focused on concrete disciplines like anatomy or unidirectional knowledge transfer, this study systematically constructs a teaching model in the highly abstract and mechanistically complex field of immunology. It is uniquely designed to deeply integrate microscopic molecular mechanisms (e.g., complement cascades) with macroscopic clinical decision-making (e.g., diagnosis and treatment of autoimmune diseases), thereby addressing the discipline’s long-standing core challenge of the theory-practice disconnect.
(2) Different teaching models: A closed-loop, data-driven pedagogical framework. Unlike traditional Computer-Assisted Instruction (CAI), which relies on preset question banks or static cases, this study constructs a complete teaching loop consisting of “clinical problem identification →AI solution generation → teaching implementation → effectiveness verification → dynamic iteration.” This model, driven by generative AI, achieves dynamic optimization based on real-time teaching feedback data, reducing the teaching iteration cycle from the traditional several months to just a few days. This represents a transformative shift in the teaching paradigm from “experience-driven” to “data-driven.”
(3) Different experimental data generation: Multi-dimensional evaluation of cognitive and clinical outcomes. Unlike existing research that primarily focuses on knowledge mastery (grades), this study employs a multidimensional mixed-methods approach for effect validation. The quantitative data not only include knowledge mastery (Host-Pathogen Interactions Concept Inventory, HPI-CI) but also incorporate clinical decision-making ability (Clinical Decision-Making in Nursing Scale, CDMNS), cognitive load (NASA Task Load Index, NASA-TLX), and knowledge retention rates. Qualitative data, on the other hand, delves into students’ acceptance and changes in learning experiences through the Unified Theory of Acceptance and Use of Technology (UTAUT) model and thematic analysis, thereby providing a comprehensive and nuanced evaluation of the model’s effectiveness.

## 2 Literature review

### 2.1 Theoretical foundation: the 4C/ID model in complex learning

Current medical education is undergoing a paradigm shift from knowledge transmission to competency-based approaches, providing a theoretical foundation for technological innovation. The 4C/ID (Four-Component Instructional Design) instructional model centers on the construction of complex learning environments, emphasizing the integration of cognitive skills, practical operations, and metacognitive abilities through task decomposition, progressive design, and the provision of supportive information within authentic tasks. This approach aligns closely with the demand for a deep integration of theoretical knowledge and clinical practice in clinical immunology education. The 4C/ID model has been shown to significantly enhance learners’ mastery of skills in complex skill training, such as clinical diagnosis (Van Merriënboer et al., 2002; Frerejean et al., 2023). Implementing the 4C/ID model in medical education, through holistic task learning and progressively complex instructional design, aids in improving learners’ clinical reasoning abilities, particularly in handling complex, multifactorial cases (Vandewaetere et al., 2015). The holistic task learning and scaffolding principles of the 4C/ID model are particularly suited to immunology education, as they can theoretically bridge the gap between understanding microscopic immune mechanisms (e.g., T-cell activation cascades) and making macroscopic clinical decisions (e.g., designing an immunotherapy regimen).

### 2.2 Cognitive load theory and the potential of dynamic instructional design

The application of Cognitive Load Theory (CLT) in medical education provides an important perspective for optimizing instructional design. Sweller et al. (2019) argue that due to the involvement of a large amount of complex information in medical education, instructional design should manage extraneous cognitive load by optimizing the presentation of information and task structure, while also enhancing germane cognitive load to improve learning efficiency. A meta-analysis by Yammine and Violato (2015) indicates that multimedia learning in anatomy education slightly outperforms traditional methods in immediate knowledge acquisition (effect size + 0.28), but does not show a significant advantage in long-term knowledge retention. Similarly, research by Cevallos et al. (2022) demonstrates that the use of virtual reality (VR) technology in orthopedic surgical training effectively enhances trainees’ spatial cognition and operational precision regarding complex anatomical structures. While these studies optimized static content, the advent of generative AI offers a paradigm shift. Its ability to dynamically generate and adjust instructional materials in real-time based on learner performance presents a novel opportunity to achieve the dynamic regulation of cognitive load called for by CLT, a potential that remains largely unexplored in immunology education.

### 2.3 The evolving role of AI and generative AI in medical education

Generative Artificial Intelligence (Generative AI), particularly represented by the advancements in Large Language Models (LLMs), is transforming higher education. Recent systematic reviews and empirical studies (Karabacak et al., 2023; Preiksaitis and Rose, 2023; Batista et al., 2024; Gumilar et al., 2025) highlight its core value in educational contexts primarily in two aspects: first, its efficient content generation capabilities, which can produce exercises on demand, distill complex knowledge points, and simulate conversational scenarios; second, its personalized interactive dialogue features, which provide technological possibilities for adaptive tutoring. These capabilities position Generative AI as a powerful tool for enhancing student engagement and supporting the acquisition of factual knowledge.

However, when focusing on the field of medical education, which requires the cultivation of advanced clinical reasoning and complex problem-solving skills, existing research tends to be cautious in evaluating the application effects of generative AI. Current evidence indicates significant shortcomings in the use of generative AI within medical education. Its primary limitation lies in its ability to effectively provide information or even generate differential diagnoses, while demonstrating insufficient efficacy in promoting the development of core competencies such as clinical reasoning and critical thinking (Lakshan et al., 2024; Göndöcs and Dörfler, 2024). Furthermore, the outputs of generative AI often lack a deep deconstruction of clinical problem contexts, making it difficult to transparently present the logical pathways from clinical manifestations to final decisions, which is crucial for fostering mature clinical thinking (Krishnan et al., 2023).

Currently, the application of generative AI in education primarily focuses on optimizing the efficiency of knowledge transmission. However, it has not fundamentally established a teaching framework that systematically fosters the progressive development of clinical thinking. A key direction for future research is to deeply integrate generative AI into a competency-based teaching framework characterized by rigorous instructional design, such as the 4C/ID model. This integration aims to address the structural deficiencies in cultivating higher-order thinking skills.

In summary, the literature reveals three critical gaps. First, the application of the 4C/ID model in immunology remains theoretical, lacking operational frameworks (Vandewaetere et al., 2015). Second, cognitive load optimization strategies have not yet leveraged the dynamic capabilities of generative AI (Sweller et al., 2019). Third, while AI tools show promise in knowledge acquisition, their efficacy in fostering integrated clinical decision-making in complex disciplines like immunology is unproven (Krishnan et al., 2023). These gaps collectively highlight the need for a new model that synergizes pedagogical theory, dynamic AI generation, and comprehensive evaluation.

## 3 Construction of IAIE model

### 3.1 Theoretical framework and model design

Based on the aforementioned theory, this study constructs an Iterative AI-Enhanced Immunology Education (IAIE) model, aimed at synergistically applying the 4C/ID model and CLT in teaching practice through generative artificial intelligence. In this model, the 4C/ID framework provides a core instructional design blueprint for organizing teaching activities: generative AI is utilized to decompose complex clinical cases in real-time into specific “learning tasks”, and automatically generate corresponding components of “relevant knowledge”, “procedural support”, and “targeted practice”. Meanwhile, CLT serves as a dynamic guiding principle for regulating the implementation of teaching: the AI engine monitors teacher-student interactions and task completion times in real-time, dynamically adjusting the complexity of tasks, the granularity of prompts, and the presentation of content to ensure that learners’ cognitive resources are efficiently utilized for knowledge construction rather than wasted on redundant information processing.

**Figure 1:**
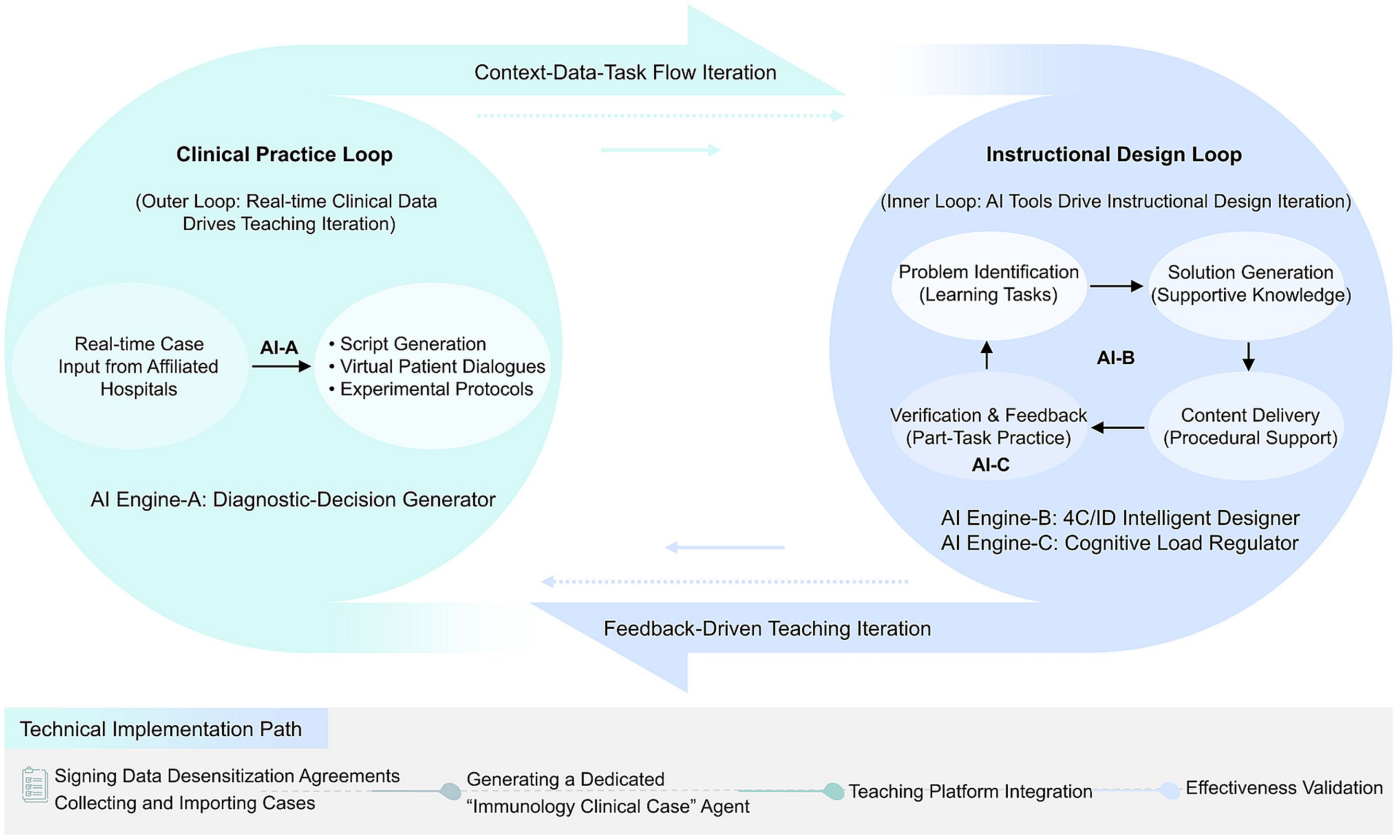
IAIE model diagram.

This “design-regulation” collaborative mechanism is realized through a dual-loop structure. The structured design of the 4C/ID model ensures the systematic and comprehensive nature of teaching, organically linking micro-level immunological mechanisms with macro-level clinical decision-making; while the CLT-driven dynamic regulation guarantees the controllability and personalization of the learning process, preventing cognitive overload when dealing with complex immunological concepts. Consequently, the theoretical synergy is transformed into a functional and iterative teaching framework, as illustrated in Figure 1.

### 3.2 The “clinical-teaching” dual-loop coupling mechanism

The core operation of the IAIE model is a “clinical-teaching” dual-loop structure driven by generative AI, as illustrated in Figure 1. This framework aims to establish a seamless and dynamic bridge between real clinical practice and structured learning.

The clinical real-world task loop is driven by real-time case data from affiliated hospitals. Within this loop, AI Engine A (a diagnostic-decision generator built on the Retrieval-Augmented Generation + Large Language Model framework) serves as the real-time case driver. It can instantly transform the input clinical data into immersive learning resources, including detailed clinical case scripts, interactive virtual patient dialogues, and suggested experimental protocols. This ensures that the learning content remains consistently aligned with current medical practices.

At the same time, the instructional design cycle transforms these authentic clinical resources into learning experiences that align with pedagogical principles. AI Engine B (4C/ID Intelligent Designer) automatically deconstructs AI-generated cases into the four components of the 4C/ID model: learning tasks, relevant knowledge, procedural support, and practice exercises. Operating within this cycle, AI Engine C (Cognitive Load Regulator) monitors real-time interactions and student response times in the classroom. Based on this data, it dynamically adjusts task complexity, the granularity of prompt information, and content presentation modes to prevent cognitive overload and optimize learning efficiency.

The model’s updating mechanism is driven by continuous, data-driven iterations between two cycles. The teaching process does not conclude upon the completion of a pre-designed task. Instead, the data generated from student performance and engagement during the instructional design cycle—such as diagnostic reasoning pathways, interaction logs, and assessment results—are fed back to the AI engine. This feedback, in turn, guides AI engine A in generating subsequent clinical cases and optimizes the instructional support provided by engines B and C. This creates a closed-loop system in which teaching is continuously refined based on empirical evidence, allowing the entire instructional process to dynamically evolve according to the needs of the learners.

### 3.3 Technical implementation path

**Figure 2:**
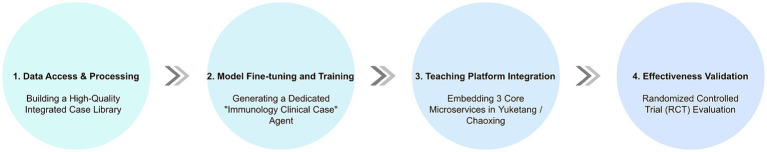
Technical implementation path of IAIE.

The implementation of the IAIE model is carried out through four structured steps, transforming the theoretical double-loop framework into a functional teaching system (Figure 2).

Step 1: Data acquisition and curation. A data de-identification protocol was established with the information department of the affiliated hospital to ensure the secure and real-time input of anonymized immunology-related clinical cases. Additionally, supplementary case studies were obtained from online clinical databases (such as PubMed and UpToDate) to broaden the diversity and scope of the teaching case repository, ensuring a rich and varied pool of authentic clinical problems.

Step 2: Model fine-tuning and tool selection. To drive a specialized AI engine, we developed a dedicated “Clinical Immunology Case Intelligent Agent”. This involved fine-tuning a foundational large language model using a carefully curated corpus that includes historical cases and immunology textbooks from the past 5 years. We strategically selected a suite of generative AI tools based on their unique functionalities to specifically implement the various components of the model, with detailed correspondences outlined in Table 1.

**Table 1:** Teaching adaptability of AI tools.

Tool	Functionality	Typical application scenarios	Educational value
ChatGPT, Grok	Question generator	Creating progressive clinical question chains	Stimulating cognitive conflict
DeepSeek, Kling AI	Knowledge deconstruction engine	Generating dynamic visual content of molecular mechanisms	Reducing intrinsic cognitive load
Gemini	Collaboration platform	Designing multidisciplinary virtual consultation tasks	Cultivating clinical decision-making skills

Step 3: Platform integration and service embedding. The fine-tuned model is deployed as independent microservices alongside selected AI tools within existing educational platforms (such as Xuetangx or Chaoxing). We have integrated three core services: a case generation service (corresponding to AI Engine A, driven by ChatGPT/Grok), a 4C/ID instructional design service (corresponding to AI Engine B), and a cognitive load monitoring service (corresponding to AI Engine C). This microservice architecture ensures modularity, scalability, and seamless integration with the platform’s existing learning management functionalities.

Step 4: Effect verification and iterative feedback. A randomized controlled trial design was employed to rigorously evaluate the effectiveness of the model. As described in the methodology section, the experimental group utilized the complete IAIE model for learning, while the control group received traditional lecture-based instruction. Key indicators for validation included diagnostic reasoning scores, accuracy rates on transfer tasks, and cognitive load scale ratings. The results from this validation phase (detailed in research methods) were subsequently used to guide the iterative optimization of the AI model and instructional design, thereby closing the loop of the IAIE framework.

### 3.4 Exemplary AI application scenario: “hyperacute rejection in organ transplantation”

To illustrate the teaching implementation process of the IAIE model, this paper uses the theme of ‘hyperacute rejection in organ transplantation’ as an example to detail its instructional workflow. This scenario demonstrates how generative AI can realize the four components of the 4C/ID model while adhering to the principles of CLT.

The learning task segment is initiated by AI Engine A (the diagnostic-decision generator). This engine dynamically constructs clinical cases based on real-time kidney transplant data, integrating patient information, laboratory dynamic curves, and disease timelines. The complexity of the cases is automatically adjusted according to students’ pre-test scores, ensuring that intrinsic cognitive load is appropriately managed and presenting a controllable level of challenge.

During the relevant knowledge phase, AI Engine B (the 4C/ID intelligent designer) automatically generates interactive, collapsible concept maps that integrate concepts at the molecular, cellular, organ, and clinical levels. Students can inquire about any node using natural language (e.g., “Why is IgM particularly effective in activating complement?”) and receive concise explanations with examples. This visualization and on-demand information retrieval significantly reduce extraneous cognitive load, allowing learners to focus on understanding conceptual relationships rather than searching for fragmented information.

Support for the program is provided by AI Engine C (the cognitive load regulator) during subsequent virtual simulation experiments. When students enter the virtual ‘complement hemolysis experiment’, the system pushes micro-guidance cards (occupying no more than 1/4 of the screen) that contain only 3–4 key steps or numerical thresholds. These cards automatically disappear after a 30-s countdown. As students’ operational proficiency increases, these prompts will gradually decrease. This scaffolding strategy optimizes associative cognitive load by freeing up working memory resources, enabling students to concentrate on core decision-making and facilitating the transition from external guidance to internal automation.

Finally, targeted practice is achieved through the AI question bank engine. If a student performs poorly in calculating complement titers, the system automatically generates a series of isomorphic practice questions with varying numerical values and clinical scenarios. Distributed practice will continue until the student’s accuracy exceeds 90% and response time is under 2 min. This targeted training promotes skill automation, freeing up working memory resources for higher-order clinical decision-making tasks, thereby enhancing overall problem-solving abilities.

## 4 Research methods

### 4.1 Research design and participants

This study employed a 12-week quasi-experimental design with a pre-test and post-test control group to evaluate the effectiveness of the IAIE model. The research received ethical approval from the Ethics Committee of Henan University (Approval No: HUSOM2025-925) and obtained written informed consent from all participants. The experimental data were anonymized, adhering to the ethical standards for medical education research outlined in the Declaration of Helsinki.

Participants included 177 undergraduate students enrolled in the Medical Immunology course at Henan University (mean age = 19.4 ± 0.7 years; 84 males and 93 females). Using a random number table method, participants were assigned to either the experimental group (*n* = 88, receiving IAIE model instruction) or the control group (*n* = 89, receiving traditional lecture-based instruction). Independent samples *t*-tests and chi-square tests confirmed the equivalence of the two groups at baseline, with no significant differences in entrance examination scores (*t* = 1.32, *p* = 0.19) and average GPA from prerequisite courses (*χ*^2^ = 2.07, *p* = 0.15).

### 4.2 Intervention procedure and data collection

This study’s teaching intervention lasted for 12 weeks and covered three thematic units: introduction to immunology, basic immunology, and clinical immunology. The experimental group employed a blended learning model consisting of “online IAIE pre-study + offline classroom deepening”. Before each formal lecture, students were required to complete a standardized learning cycle on the IAIE platform, which included: (1) AI-generated clinical case analyses, (2) scaffolded concept exploration using interactive knowledge graphs, (3) virtual laboratory simulations with adaptive prompts, and (4) automated specialized exercises with immediate feedback. In the subsequent offline classes, instructors did not repeat the explanation of foundational knowledge; instead, they focused on addressing common difficulties identified through the learning data analysis provided by the IAIE platform. This included targeted explanations and organizing group discussions for deeper case analysis and decision-making exercises. The control group followed a “traditional lecture + homework” model, receiving systematic instruction on the same content in class and completing an equivalent amount of conventional exercises afterward.

Data collection adhered to a structured timeline: pre-assessment (including Knowledge Proficiency Index-Cognitive Integration, Clinical Decision-Making Ability, and NASA-TLX Cognitive Load Scale) was conducted in week 1. The intervention was implemented from weeks 2 to 11, during which formative assessment data were continuously collected via the AI platform. Post-assessment (using a parallel version of the pre-assessment tests) took place in week 12, followed by focus group interviews (*n* = 18) and UTAUT questionnaire surveys. A delayed post-assessment to evaluate knowledge retention was conducted 4 weeks after the intervention concluded.

### 4.3 Measurement tools

The level of knowledge mastery was measured using HPI-CI (Marbach-Ad et al., 2010), which consists of 25 multiple-choice questions and has demonstrated good reliability (Cronbach’s *α* = 0.87) and content validity (CVI = 0.93). Clinical decision-making ability was assessed using an adapted version of CDMNS (Duarte and Dixe, 2021), which was deemed suitable for immunological contexts through expert review (CVI = 0.90) and exhibited good internal consistency (*α* = 0.85). Cognitive load was measured using NASA-TLX, a six-dimensional assessment scale with established psychometric properties in educational contexts (Hart, 2006). Learning experience was evaluated using a questionnaire designed based on the UTAUT (Venkatesh et al., 2003), which included 24 items across four dimensions, such as performance expectancy and effort expectancy, rated on a 5-point Likert scale. Confirmatory factor analysis indicated good model fit (CFI = 0.94, RMSEA = 0.06).

### 4.4 Data analysis

Quantitative data were subjected to analysis of covariance (ANCOVA), controlling for pre-test scores to adjust for baseline differences. Effect sizes were reported using Cohen’s d. For qualitative data from focus group interviews, thematic analysis followed the six-phase framework by Braun and Clarke (2006). The coding process was conducted using NVivo 12 software, and reliability was ensured through independent coding by two researchers (inter-coder agreement = 87%) and member checking with participants.

## 5 Results

### 5.1 Classroom performance

A 12-week experimental study demonstrated significant improvements in three key dimensions—knowledge mastery, clinical diagnostic ability, and learning efficiency—when comparing the IAIE model to traditional teaching methods (Table 2).

**Table 2:** Comparison of key educational indicators between the experimental group (IAIE model) and the control group (traditional teaching model).

Indicators	Experimental group (M ± SD)	Control group (M ± SD)	Statistical test results	Effect size
Standardized test scores	82.7 ± 6.3	70.1 ± 7.5	*F* = 28.34, *p* < 0.001	*η*^2^ = 0.13
Clinical decision accuracy (%)	81.2 ± 8.1	61.6 ± 10.3	*χ*^2^ = 19.02, *p* < 0.001	OR = 2.67
Problem-solving time (min)	8.7 ± 2.1	15.1 ± 3.8	*t* = 12.47, *p* < 0.001	Cohen’s d = 1.4
Total cognitive load score	42.1 ± 7.2	52.2 ± 9.6	*t* = 6.89, *p* < 0.001	d = 0.93

Knowledge mastery: Analysis of standardized test scores revealed that the experimental group (*n* = 88) achieved a post-test average score increase of 23.4% in foundational knowledge concepts (82.7 ± 6.3 vs. 67.0 ± 8.1, Cohen’s d = 1.12), significantly outperforming the control group’s improvement of 8.2% (70.1 ± 7.5 vs. 64.8 ± 7.9, d = 0.34). Covariance analysis confirmed that the inter-group differences were statistically significant (*F* (1,174) = 28.34, *p* < 0.001, *η*^2^ = 0.13). In tests assessing higher-order mechanisms (such as T cell activation thresholds and complement cascade regulation), the experimental group’s accuracy reached 78.9%, surpassing the control group’s accuracy of 52.4% by 26.5 percentage points (*χ*^2^ = 27.83, *p* < 0.001). Confusion matrix analysis of pre- and post-tests indicated that the experimental group’s misunderstanding rate of the ‘immune checkpoint inhibition mechanism’ decreased from 34% to 9%.

Clinical ability: assessment using the adapted CDMNS scale indicated that the experimental group improved their diagnostic accuracy in autoimmune disease differential diagnosis by 31.7% (81.2% vs. 61.6%). Error type analysis revealed a shift in the primary causes of misdiagnosis: the control group’s main error was attributed to “mechanism understanding deviation” (63%), while the experimental group’s primary challenge shifted to “insufficient clinical manifestation recognition” (37%). In a complex case scenario involving HIV with cryptococcal meningitis, 89% of students in the experimental group correctly identified “intracranial hypertension and brain herniation” as key complications, demonstrating enhanced crisis management skills. However, the assessment also highlighted students’ relative weaknesses in prioritising integrated decision-making. For instance, in the case of systemic lupus erythematosus (SLE), the decision-making logic regarding hormone shock versus correcting acidosis tended to reflect fragmented memory of treatment measures.

Learning efficiency: measurements using the NASA-TLX scale indicated that the experimental group’s intrinsic cognitive load (“mental demand” + “effort level”) decreased by 19.3% (M = 42.1 vs. 52.2), while their associated cognitive load (“self-performance” + “effort level”) increased by 22.7% (M = 68.5 vs. 55.8), suggesting that AI tools facilitated effective reallocation of cognitive resources (*t* = 5.89, *p* < 0.001). Delayed post-test results (4 weeks later) further revealed that the experimental group’s knowledge retention rate for core concepts (74.3%) was significantly higher than that of the control group (51.8%) (Table 2).

### 5.2 Teaching recognition survey

**Figure 3:**
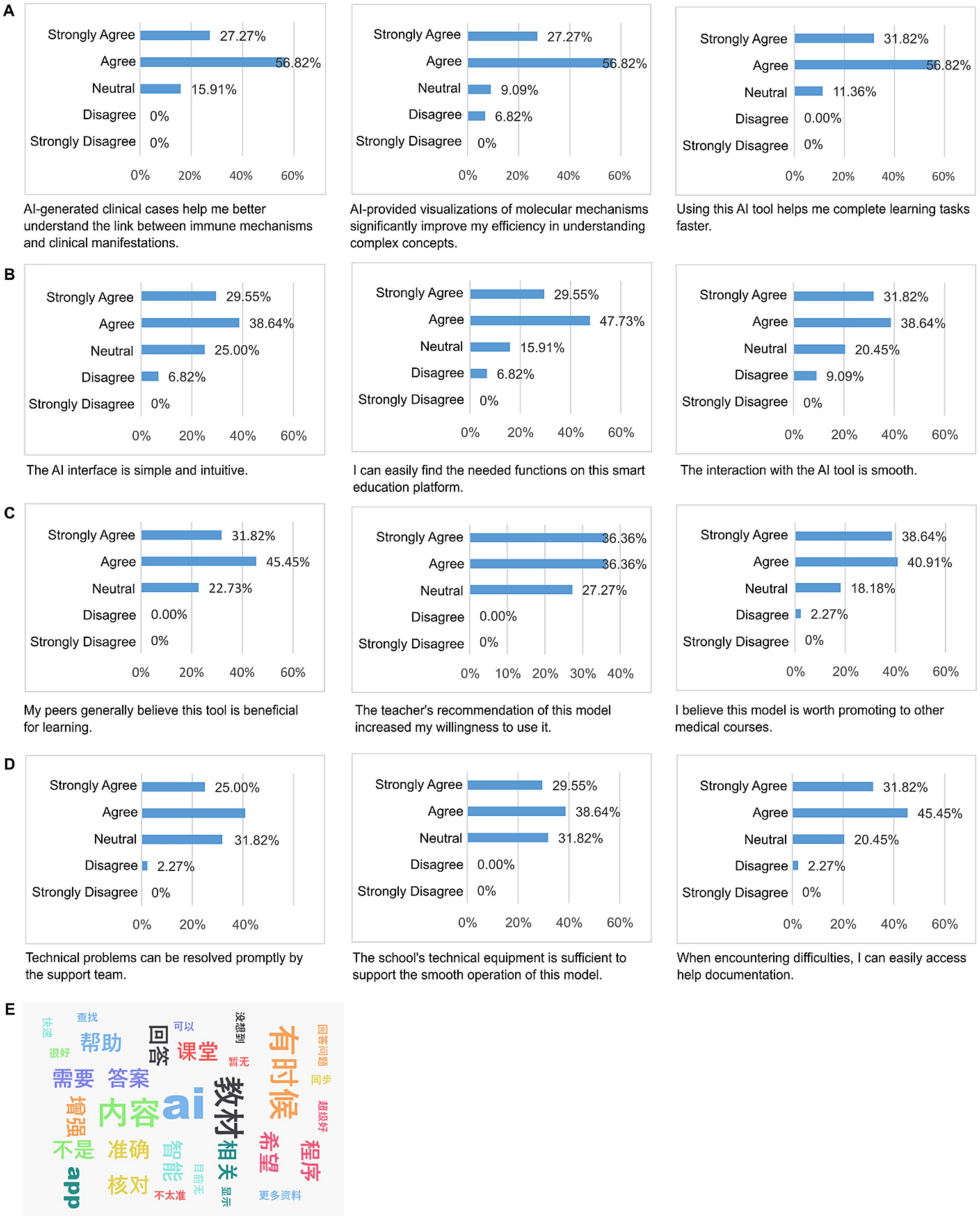
UTAUT four-dimensional scoring radar chart showing: **(A)** Performance expectation, **(B)** Effort expectation, **(C)** Social influence, **(D)** Facilitating conditions, **(E)** Social influence word cloud.

The results from the questionnaire survey (UTAUT model, *n* = 88) and focus group interviews (3 groups, *n* = 18) indicate that students have a high acceptance of the IAIE model (Figure 3).

Quantitative analysis reveals the following findings: (1) Performance expectancy: 84.09% of students agreed or strongly agreed that AI-generated clinical cases (such as SLE cases) aid in understanding the relationship between immune mechanisms and clinical manifestations. Additionally, 84.09% of students recognized the value of AI visual content (such as antigen presentation animations) in enhancing understanding efficiency. Furthermore, 88.64% of students believed that AI tools significantly accelerated the completion speed of learning tasks. (2) Effort expectancy: 68.19% of students found the ‘Rain Classroom Intelligent Learning Companion’ interface to be intuitive and user-friendly; 77.28% of students could quickly locate the required functions; and 70.46% of students felt that the interaction process with AI tools was smooth. (3) Social influence: 77.27% of students observed that peers generally recognized the value of this tool; 72.72% of students reported an increased willingness to use the tool due to recommendations from teachers; and 79.55% of students supported the promotion of the IAIE model to other medical courses (such as pathology). (4) Facilitating conditions: 65.91% of students believed that technical issues on the platform (such as system delays) could be promptly resolved by the support team; 68.19% of students felt that the school’s technical equipment (such as network/hardware) was sufficient to support the smooth operation of the model; and 77.27% of students found it easy to access help documentation when encountering operational difficulties.

Qualitative thematic analysis (NVivo 12) identified three core themes closely related to the AI-driven 4C/ID instructional mechanism. At the level of “Complex Concept Analysis” (node coverage 34.7%), students generally reported that the AI’s use of plain language and immediate examples transformed abstract mechanisms (such as TI antigens and complement cascade reactions) into perceivable processes, thereby reducing internal cognitive load (e.g., “AI explained BCR cross-linking using the analogy of ‘multiple keys opening a lock simultaneously’…”). Regarding the enhancement of learning efficiency (node coverage 29.1%), students noted that AI-generated situational summaries and exam highlights shortened information retrieval time, allowing them to allocate more cognitive resources to solving complex problems and making clinical decisions, thus demonstrating higher cognitive efficiency (e.g., “AI simply explained mechanisms with examples, making the immune response process clearer.”). In terms of resource integration needs (node coverage 18.4%), respondents expressed a desire for the interface to simultaneously display textbook content and enhance the academic authority of literature sources (Figure 3E) (e.g., “I hope AI can display textbook content simultaneously; the authority of literature searches needs to be strengthened.”). These findings provide clear directions for subsequent system iterations and validate the feasibility of AI in regulating complex learning loads.

### 5.3 Effect evaluation

Evaluation using the IAIE-reflective practice scale (IAIE-RPS) (Rogers et al., 2024) and the Dundee Ready Education Environment Measure (DREEM) (Roff et al., 1997) scale for the experimental group (*n* = 85/89) yielded positive results.

The results of the IAIE-RPS indicate that students exhibit a high level of reflective practice ability, with an average total score of 5.45 out of 7. Key findings include: effective stimulation of higher-order thinking, particularly in the area of clinical decision-making reflection (M = 5.69); steady development of metacognitive skills, including the adjustment of learning plans based on AI feedback on learning trajectories (M = 5.73), as well as self-correction when AI responses conflict with personal judgments (M = 5.67); and the preliminary formation of critical validation awareness, where students consciously assess the reliability of AI-generated content and compare it with textbooks and literature (M = 5.85) (Table 3).

**Table 3:** Means for IAIE-RPS.

Reflecting on the practical dimension	Number of items	Mean	Stand deviation	*t* value	*p*-value
Higher-order thinking activation	4	5.45	0.78	5.32	<0.001
Metacognitive ability development	4	5.69	0.65	6.11	<0.001
Critical verification awareness	4	5.85	0.71	7.02	<0.001
Total meter	12	5.67	0.59	6.45	<0.001

**Figure 4:**
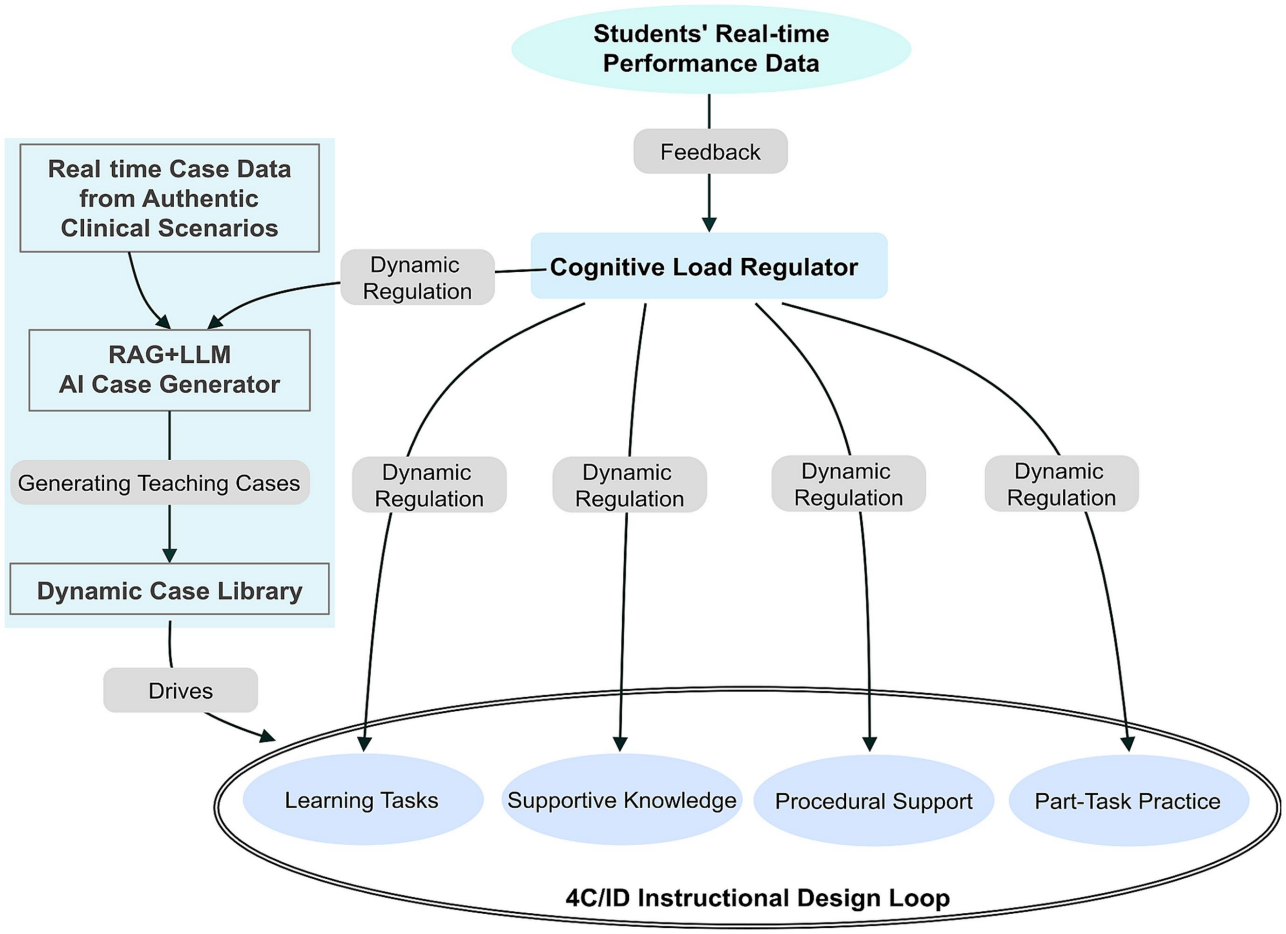
“Clinical-teaching” dual-cycle coupling concept model of the IAIE model.

The analysis of the DREEM scale indicates that the IAIE scores significantly exceed the benchmarks of traditional teaching environments, demonstrating that students highly recognize the learning environment created by IAIE. There is an enhancement in academic self-efficacy: 95.6% of students feel more confident in solving clinical immunology problems and acknowledge an improvement in their clinical reasoning abilities. The role of teachers has successfully transformed: 96.7% of students recognize that teachers act more as “facilitators or coaches” in the new model, encouraging the use of AI for independent exploration. The learning environment and technological support are robust: 91.1% of students believe that the school’s physical facilities (such as internet access) facilitate the smooth implementation of AI-assisted learning, and 93.3% of students feel that the collaborative features on the AI platform enhance academic communication among peers. A deeper analysis of each core area further reveals that while the learning environment maintains overall high quality, there remains clear room for optimization in aspects such as learning pace and academic opportunities (Tables 4, 5, and Figure 4).

**Table 4:** Overall assessment results of the DREEM scale in the IAIE learning environment (*N* = 90).

Core area	Title number	Average score in the field (out of 4)	Overall performance evaluation
Perception of learning by students (SPL)	1–3	3.45	Very good
Perception of teachers by students (PTS)	4–7	3.56	Very good
Academic self-perception of students (ASPS)	8–10	3.46	Very good
Perception of the environment by students (PES)	11–13	3.16	Better (with room for improvement)
Social self-perception of students (SSPS)	14–16	3.35	Very good
Overall quality of the educational environment	1–16	3.40	Very good

The performance evaluation refers to the general standards of the DREEM scale (0–2 points: many issues present; 2–3 points: needs improvement; 3–4 points: very good).

**Table 5:** Key focus of teaching improvement based on DREEM scale results.

Improvement dimensions	Specific issues (item number and core description)	Key data support	Teaching improvement suggestions
Learning pace and stress management	11. The learning pace of the course is comfortable and sustainable	The average score is only 2.68; 18.9% of students hold negative opinions (disagree + strongly disagree)	Examine the course capacity and schedule, considering increasing flexibility, such as providing differentiated learning paths or tasks to alleviate student stress.
Extracurricular academic opportunities	16. Opportunities to participate in extracurricular academic activities in immunology	The average score of 3.23 is relatively low; 17.8% of students expressed uncertainty	Proactively organize or promote extracurricular activities such as lectures, seminars, and knowledge competitions, linking them with course content to enhance student engagement and academic immersion.
Focus of course content	2. The course places more emphasis on understanding application rather than rote memorization	The average score is 3.13; 11.1% of students explicitly stated ‘strongly disagree’	Further strengthen the assessment of understanding immunological mechanisms and clinical application abilities in case design and evaluation methods, and clearly communicate this teaching philosophy to students.

### 5.4 Explanation of anomalous data

A small portion of students (7.2%, *n* = 7) initially experienced adaptation difficulties, primarily due to technology anxiety (*n* = 3, concerns about operational errors affecting grades) or cognitive overload when faced with multimodal content (*n* = 4). After implementing interventions such as additional guidance modules (e.g., an “AI Tool Operation Guide” animation) and dynamically adjusting content density, the incidence of maladaptation decreased to 1.8% by the end of the 12-week experiment.

## 6 Discussion

This study developed the IAIE model, which integrates generative artificial intelligence with the 4C/ID framework and CLT to address long-standing challenges in immunology education. A 12-week experiment demonstrated that the IAIE model significantly enhanced students’ knowledge retention, clinical decision-making accuracy, and learning efficiency compared to traditional teaching methods. Evaluations using the DREEM and IAIE-RPS scales further confirmed the high acceptance of the model among students and their strong reflective practice levels.

### 6.1 The IAIE model as a catalyst for deep learning: the integration of theory and practice

Our research findings indicate that the IAIE model facilitates deep learning by dynamically connecting theoretical knowledge with clinical practice, addressing a common challenge in medical education (Haidaris and Frelinger, 2019; Siani et al., 2024). The generative artificial intelligence-driven ‘clinical-teaching’ dual-cycle coupling mechanism provides a continuous flow of real-world tasks. This aligns with the perspective of situated cognition theory, which posits that knowledge is constructed within specific contexts (Brown et al., 1989). The significant improvement in clinical reasoning accuracy (31.7%, *p* < 0.001) suggests that engaging with these AI-generated, nearly authentic clinical scenarios aids students in developing robust pattern recognition and decision-making skills under real-world pressure.

Furthermore, the IAIE model effectively manages cognitive load, a critical factor in learning within complex domains (Sweller et al., 2019). Data from the NASA-TLX scale reveal a significant restructuring of cognitive resources: a reduction in intrinsic cognitive load and an increase in extraneous cognitive load. This indicates that AI tools, such as cognitive load regulators, successfully simplify the processing of complex immunological mechanisms, thereby freeing up working memory resources for deeper cognitive activities, such as concept integration and clinical transfer. This finding extends the application of CLT by demonstrating the feasibility of dynamic load regulation through artificial intelligence in real-time classroom environments.

Specifically addressing the pedagogical challenges of immunology, the IAIE model’s strength lies in its targeted modulation of cognitive load. For abstract concepts like the complement cascade, AI Engine C dynamically manages extraneous load by providing interactive, visual knowledge graphs (AI Engine B) and just-in-time micro-guidance, preventing students from being overwhelmed by isolated facts. Simultaneously, it optimizes germane load by presenting progressively complex, AI-generated clinical cases (AI Engine A) that require learners to actively integrate molecular mechanisms with clinical manifestations. This dynamic scaffolding, informed by Cognitive Load Theory (Sweller et al., 2019), ensures that working memory resources are directed toward the essential process of constructing mental models of immune system function and dysfunction, rather than being consumed by information search or procedural confusion.

### 6.2 Cultivating metacognition and critical thinking in an artificial intelligence-enhanced environment

A particularly promising finding is the high levels of reflective practice and critical validation awareness reported by the IAIE-RPS scale. Students not only utilize artificial intelligence for knowledge acquisition but also actively engage in metacognitive activities, questioning the outputs of AI and comparing them with textbooks and literature (M = 5.85). This suggests that, contrary to concerns about AI fostering passive learning (Lakshan et al., 2024), the IAIE model can cultivate academic discernment. This aligns with Zimmerman’s (2002) self-regulated learning model, where AI serves as a source of immediate feedback, enabling students to monitor and adjust their learning pathways and clinical reasoning processes.

Social network analysis indicates a higher interaction density within the experimental group, highlighting the model’s role in creating a distributed cognitive space. The environment fostered by this AI-driven collaborative platform encourages knowledge sharing and peer dialogue, resonating with Vygotsky’s (1978) social development theory. Therefore, the IAIE model appears to support a holistic cognitive development ecosystem, where individual metacognition synergizes with social learning to promote deep understanding.

### 6.3 Beyond traditional educational technology: from static content to dynamic interaction

The innovation of the IAIE model is evident when compared to traditional computer-assisted instruction (CAI). Traditional CAI typically relies on static, predefined content and fixed question banks, whereas the generative AI in IAIE allows for the creation of dynamic content that evolves alongside scientific advancements. This shift from static curricula to dynamic learning experiences represents a significant paradigm change, ensuring that students engage with the latest clinical data and research trends in real time.

Furthermore, the transition from menu-based interactions in traditional CAI to natural language dialogues with AI tools marks a major advancement in instructional delivery. This not only enhances the complexity and authenticity of student inquiries—better reflecting the open-ended nature of clinical questioning—but also promotes higher-order thinking. According to Bloom’s taxonomy, the observed increase in cognitively demanding tasks underscores the potential of generative AI to engage students in professionally relevant, authentic problem-solving.

While recent studies have explored generative AI in medical education, many focus on its role as an information repository or for generating basic quizzes (Karabacak et al., 2023; Preiksaitis and Rose, 2023). In contrast, our IAIE model’s innovation lies in its deep integration with the 4C/ID pedagogical framework. Unlike studies that simply assess AI’s accuracy in answering immunology questions, our work provides a structured pathway for learners to apply AI-generated knowledge within a holistic task context. Furthermore, compared to other AI applications that offer static content, the dynamic, feedback-driven regulation of cognitive load by our model represents a significant advancement, actively adapting the learning process in real time rather than merely delivering information.

### 6.4 Practical significance, acknowledging limitations and future directions

The practical significance of this study lies in providing a transferable artificial intelligence integration model for medical education, detailing tool selection, content review, and effectiveness evaluation. The successful interdisciplinary validation within our institution indicates that this model has potential applications beyond immunology.

While this study provides robust evidence of the IAIE model’s efficacy within our specific context, it is important to acknowledge the limitations that may affect the generalizability of the findings. The substantial effect sizes observed (e.g., Cohen’s d = 1.12 for knowledge mastery), achieved under controlled conditions, underscore the potential of this theory-driven IAIE model to meaningfully enhance learning within a single semester. However, these promising results should be interpreted in light of the following limitations.

First, the sample was drawn from a single university, which may affect the generalizability of the findings. While the results obtained within this specific context are promising, they may not fully capture the diversity of institutional contexts or student populations. Future multi-center studies involving students from institutions with varying academic profiles are necessary to confirm the model’s broad applicability.

Second, the study’s temporal scope presents additional limitations. The delayed post-test was conducted only 4 weeks post-intervention. This timeframe, while aligned with the conclusion of a single academic semester and useful for assessing short-term knowledge retention, is insufficient to evaluate the model’s long-term effectiveness or its true impact on students’ subsequent clinical performance. Furthermore, the 12-week intervention period, though consistent with the natural pacing of the semester-based curriculum and sufficient to observe significant learning gains, cannot capture the model’s sustained effects on professional competency development. Future studies should incorporate longer follow-up periods, ideally tracking students into their clinical clerkships to assess the durability and transfer of learning outcomes.

Third, while the model significantly improved foundational knowledge and clinical reasoning, the development of advanced and nuanced clinical judgment skills requires further systematic support and extended training periods. Future research should investigate the design of AI-driven adaptive scaffolding and longitudinally integrated case-based simulations to systematically foster these higher-order competencies over extended training periods.

To address these limitations and extend the model’s impact, future work will focus on: (1) expanding the AI-curated library of immunology-related disease models to encompass a broader range of pathophysiological scenarios; (2) enhancing the integration of AI with textbooks and literature to improve content authority and student trust; and (3) exploring multimodal interactions by integrating students’ learning task completion paths and cognitive load data within the platform to further optimize the learning process and personalization.

## 7 Conclusion

This study constructs and validates an IAIE model, providing theoretical support and practical solutions for innovations in medical education. By organically integrating generative artificial intelligence with the 4C/ID instructional design model and CLT, this model effectively addresses core issues that have long plagued immunology education, such as the disconnection between theory and practice and difficulties in understanding complex concepts.

Empirical research indicates that the IAIE model achieves dynamic optimization of the teaching process through a ‘clinical-teaching’ dual-loop mechanism (Figure 4). Compared to traditional teaching methods, this model demonstrates significant advantages in enhancing students’ knowledge retention (an increase of 23.4%) and clinical decision-making abilities (an increase of 31.7%). Notably, while maintaining the advantages of technological empowerment, the model successfully fosters a critical attitude among students towards AI-generated content, a trait that holds significant importance for the future training of medical professionals.

The theoretical value of this research lies in expanding the application boundaries of the 4C/ID model within intelligent educational environments and providing empirical evidence for dynamic cognitive load regulation. On a practical level, the transferable AI education implementation plan offered by this study—including technology integration pathways, content review mechanisms, and effectiveness evaluation systems—serves as an important reference for educational innovations in related fields.

Future research will focus on advancing the following directions: enhancing the breadth and depth of the disease model database, establishing effective validation mechanisms for AI-generated content against authoritative teaching resources, and developing learning analytics systems based on multimodal data. These efforts will further enhance the educational value of the IAIE model and promote the development of medical education towards a more personalized and precise approach.

## Data Availability

The original contributions presented in the study are included in the article. Further inquiries can be directed to the corresponding author.
